# Health workforce readiness for future pandemics: Evidence from undergraduate health sciences students in Georgia

**DOI:** 10.3934/publichealth.2026044

**Published:** 2026-08-06

**Authors:** Dea Goderdzishvili, Elza Nikoleishvili

**Affiliations:** Public Health and Healthcare Management Department, School of Health Sciences, The University of Georgia, Tbilisi, Georgia

**Keywords:** pandemic preparedness, healthcare workforce, public health, undergraduate education, workforce readiness, health systems resilience, experiential learning, COVID-19, Georgia

## Abstract

**Background:**

Strengthening health workforce preparedness has become a critical public health priority in the post-COVID-19 era, particularly in countries facing workforce shortages and uneven integration of preparedness competencies. This study evaluated self-perceived pandemic preparedness among final-year biomedical and health sciences students in Georgia and examined the role of undergraduate education in preparing students for future public health emergencies.

**Methods:**

A cross-sectional survey was conducted among final-year students from medicine, dentistry, public health, nursing, and pharmacy programs (n = 296), with nursing (n = 6) and pharmacy (n = 1) students underrepresented. An adapted questionnaire assessed self-perceived preparedness, COVID-19 response participation, willingness to engage in future public health emergencies, and educational experiences. Preparedness dimensions were identified using exploratory factor analysis, and statistical analyses included descriptive, inferential, and regression methods.

**Results:**

Overall preparedness was moderate (M = 3.50, SD = 1.14). Two preparedness dimensions were identified: theoretical, preventive, communicative, and clinical knowledge and clinical and practical readiness. Practical skills, infection control participation, and emergency response activities showed the strongest associations with overall preparedness (p < 0.001). Despite moderate preparedness, 84.1% of students expressed willingness to contribute to future public health emergencies, rising to 97.5% among those with COVID-19 response experience. Students with COVID-19 response experience demonstrated higher preparedness across domains. Clinical and medical training were identified as the highest priority for curricular reform (43.6%), surpassing preventive, theoretical, and communication-focused measures.

**Conclusions:**

A gap exists between students' willingness to contribute during public health emergencies and their self-perceived preparedness. These findings suggest that undergraduate education may play an important role in strengthening preparedness for future public health emergencies. Greater emphasis on experiential learning, simulation-based training, and supervised participation in public health activities may help improve perceived preparedness. Georgia provides a relevant case study for countries facing similar workforce constraints and fragmented integration of preparedness competencies within undergraduate education.

## 1. Introduction

The coronavirus disease 2019 (COVID-19) pandemic exposed fundamental weaknesses in global health systems and underscored the critical importance of a well-prepared health workforce. Even prior to the pandemic, a global shortage of nearly 18 million healthcare workers was projected, disproportionately affecting low- and middle-income countries [1]. During COVID-19, these shortages intensified, with widespread disruptions to service delivery and increased risks to healthcare workers [2]. In response, many countries mobilized biomedical and health sciences students to support essential services, including clinical care, public communication, and public health interventions [3]–[6]. While these efforts demonstrated the potential of students as a supplementary workforce, they also highlighted variability in preparedness and raised questions about whether undergraduate education adequately equips students for such roles.

Georgia reflects these global trends within a distinct national context. As an upper-middle-income country, it continues to face workforce shortages, uneven distribution of healthcare professionals, and structural challenges within primary care [7]. The nurse-to-physician ratio remains critically low, and regional disparities contribute to persistent gaps in access to care. During the pandemic, Georgian universities played an important role in supporting the national response, with more than 1800 final-year students rapidly trained and deployed in infection control, communication, and public health activities such as contact tracing and teleconsultations [8]. Although these initiatives demonstrated institutional adaptability, they were largely reactive and temporary, underscoring the absence of systematically embedded preparedness within undergraduate education. In contrast, several international initiatives have adopted more proactive approaches by integrating preparedness competencies into undergraduate and professional health education through dedicated preparedness programs. For example, the PREPARED initiative at Charité-Universitätsmedizin Berlin was established to strengthen pandemic preparedness and emergency response competencies through structured educational activities and interdisciplinary approaches [9]. Another example is the University of Antwerp's Pandemic Preparedness Summer School, an extracurricular European Credit Transfer and Accumulation System (ECTS)-accredited program that provides interdisciplinary training in outbreak preparedness and response for senior medical and paramedical students, while also engaging researchers, residents, and healthcare professionals [10]. Such approaches serve as longitudinal educational frameworks rather than temporary emergency response measures. Existing evidence indicates that pandemic-related competencies in Georgian biomedical and health sciences curricula remain limited and inconsistently integrated [11].

Strengthening pandemic preparedness through undergraduate education represents a critical opportunity to improve students' readiness for future public health emergencies, as they represent the next generation of health professionals. International evidence suggests that curricula combining theoretical knowledge with practical and experiential learning improve confidence, adaptability, and interdisciplinary collaboration in emergency contexts [12],[13]. However, in resource-constrained settings, limited access to structured experiential learning contributes to a persistent gap between knowledge and practical competence [14]. Prior qualitative research in Georgia has identified key areas for improvement, including simulation-based training, public health communication, and interdisciplinary collaboration, as central components for strengthening preparedness and advancing health promotion through education [15]. However, quantitative evidence examining pandemic preparedness among biomedical and health sciences students remains limited. The present study builds upon these qualitative findings by quantitatively examining students' self-perceived pandemic preparedness, their educational experiences, and readiness to contribute to future public health emergencies.

This cross-sectional study was conducted among final-year biomedical and health sciences students in Georgia and provides a timely post-COVID assessment, five years after the onset of the pandemic, of whether meaningful changes have occurred in undergraduate education and preparedness for future public health emergencies. The study examines how theoretical knowledge, practical skills, educational experiences, and prior pandemic-related exposure are associated with students' self-perceived preparedness and willingness to participate in future emergency response efforts, while also considering the potential role of students as a surge workforce during public health crises. The findings suggest that practical and experiential competencies are more strongly associated with perceived preparedness than theoretical knowledge alone, underscoring the importance of applied and practice-based learning within undergraduate education. By positioning Georgia as a case study, this research also provides insights that may be relevant to other upper-middle-income countries facing similar health workforce shortages and challenges in integrating pandemic preparedness into health professions education.

## 2. Materials and methods

### 2.1. Study design and participants

A cross-sectional study was conducted in 2024 among final-year students enrolled in medicine, dentistry, public health, nursing, and pharmacy programs who were expected to graduate in the same year. Final-year cohorts were selected because their clinical training overlapped with the COVID-19 pandemic.

Purposive sampling was used to ensure broad institutional representation across state and private universities in Georgia. Approximately 15 universities offering undergraduate biomedical and health sciences programs were contacted to support participant recruitment. Surveys were distributed through deans, administrative offices, mailing lists, and in-class sessions. Data collection was conducted between April and October 2024. As participation was voluntary, the potential for self-selection bias cannot be excluded.

Based on the national graduation data from 2023, the estimated eligible study population comprised approximately 4066 final-year students across medicine, dentistry, pharmacy, nursing, and public health programs. A total of 302 responses were collected, of which 296 were valid after data verification. The sample reflects the underlying student population but is imbalanced across disciplines, with nursing and pharmacy being under-represented. Program-level analyses were therefore restricted to medicine, dentistry, and public health, while smaller subgroups were interpreted descriptively. As recruitment occurred through multiple channels, a formal response rate could not be calculated.

#### 2.1.1. Instrument

A review of the literature on pandemic preparedness, disaster medicine, public health emergency response, and health professions education was conducted to inform questionnaire development. Relevant published surveys, educational frameworks, and pandemic assessment tools were reviewed to identify key domains and survey items. Of the 37 items in the final instrument, 20 items (Items 1, 2, 3, 4, 5, 7, 9, 10, 12, 16, 19, 21, 23, 24, 25, 27, 28, 29, 30, and 36) were adapted from constructs and items identified through this literature review, with wording modified for contextual and linguistic relevance to the Georgian educational setting. The remaining items, including demographic items and items capturing COVID-19 response experience and satisfaction, were developed specifically for this study to address objectives not comprehensively covered by existing instruments. Given the absence of a single instrument that comprehensively addressed all study objectives, the final questionnaire represented a study-specific instrument informed by and adapted from previously published literature.

The instrument contains 37 items across four sections: demographics, curriculum-based preparedness, COVID-19-related experiences and satisfaction, and perspectives on future preparedness. The preparedness domain initially included 23 Likert-scale items. Following reliability screening, three items with weak corrected item–total correlations were removed. The final scale included 20 items rated on a 5-point Likert scale (1 = strongly disagree to 5 = strongly agree; 3 = neutral), assessing perceived preparedness to respond to pandemics. Additional items included dichotomous and multiple-choice questions capturing experiences and contextual factors. Two open-ended questions were included but not analyzed due to limited responses (see Supplementary Table 1). The questionnaire was translated from Georgian into English and back-translated using standard forward–backward procedures to ensure linguistic and conceptual equivalence. For online surveys, all questions were mandatory. Paper-based responses were reviewed during data entry, and incomplete responses were excluded. Participants were instructed to complete the survey only once. Participation was voluntary. Written informed consent was obtained from all participants prior to participation, and participants who provided consent proceeded to the questionnaire section. Anonymity and confidentiality were maintained throughout. To minimize bias, recruitment was conducted across multiple institutions, question wording was kept neutral to reduce social desirability bias, and identical formats were used across online and paper surveys.

#### 2.1.2. Validity and reliability

Content validity was established through expert review and adaptation of existing instruments. Three experts reviewed the initial draft versions of the questionnaire instrument and provided feedback on item clarity, contextual relevance, and terminology, with minor revisions made to improve wording and enhance applicability to the Georgian educational context. All reviewers had backgrounds in biomedical and health sciences education and held doctoral qualifications in public health or medicine. Internal consistency was assessed using Cronbach's alpha, and items with weak corrected item–total correlations were removed prior to analysis. Sampling adequacy was evaluated using the Kaiser–Meyer–Olkin measure, and Bartlett's test of sphericity confirmed factorability. Exploratory factor analysis was conducted using principal axis factoring with direct Oblimin rotation to identify underlying dimensions. As the factor structure was derived and evaluated within the same sample, the findings should be interpreted as exploratory and not cross-validated in an independent cohort. Reliability was assessed at the factor level. Although high internal consistency suggested potential redundancy, all retained items were included to preserve conceptual coverage across domains.

#### 2.1.3. Data analysis

Statistical analyses were performed using IBM SPSS Statistics for Windows, Version 30.0 (IBM Corp., Armonk, NY, USA). Descriptive statistics summarized demographic characteristics and preparedness scores. Inferential analyses included independent-samples t-tests, one-way analysis of variance with post hoc comparisons, Pearson correlations, multiple linear regression, and binary logistic regression.

Program-level analyses were restricted to medicine, dentistry, and public health due to small subgroup sizes in nursing and pharmacy. Factor scores were standardized (z-scores) for inferential analyses. Multiple linear regression assessed associations between preparedness dimensions and overall preparedness. Binary logistic regression was used to examine predictors of willingness to participate in future pandemics and predictors of receipt of extracurricular training.

Model assumptions, including normality, homoscedasticity, linearity, and multicollinearity, were evaluated. Variables were screened for range and outliers. Six valid low-end outliers were identified (|z| > 3.0) and retained as genuine responses. Logistic regression models were assessed using omnibus tests, classification accuracy, Nagelkerke R^2^, and the Hosmer–Lemeshow test. Statistical significance was set at α = 0.05 (two-tailed), with p < 0.001 indicating very small probabilities. No missing data were present in the final analytical dataset, as all items in the online survey were mandatory, and incomplete paper-based questionnaires were excluded during data cleaning.

### 2.2. Ethics approval of research

Ethical approval for both qualitative and quantitative components was obtained from the Biomedical Research Ethics Board of the School of Health Sciences at The University of Georgia (Approval No. N:11-6461). Participation was voluntary, and written informed consent was obtained from all participants prior to participation.

## 3. Results

### 3.1. Sample characteristics

A total of 296 valid responses were retained. Most respondents were aged 18–22 years (n = 188, 63.5%), followed by 23–27 years (n = 93, 31.4%), and 27 years or older (n = 15, 5.1%). The majority were female (n = 205, 69.3%), with n = 84 (28.4%) male, and n = 7 (2.4%) preferring not to disclose. By educational program, medicine accounted for n = 194 (65.5%), dentistry for n = 86 (29.1%), public health for n = 9 (3.0%), nursing for n = 6 (2.0%), and pharmacy for n = 1 (0.3%).

### 3.2. Preparedness scores and item-level analysis

We first examined the distribution of responses to the single-item measure assessing students' perceived preparedness to respond to pandemics. Responses were approximately moderate, with an overall mean of 3.50 (SD = 1.14, 95% CI [3.37, 3.63], range = 1–5, n = 296). Based on this item, 18.9% of respondents considered themselves unprepared, 23.6% were neutral, and 57.4% reported being prepared to some degree. Descriptive analysis of the 20 preparedness items revealed moderate to moderately high self-perceived readiness across domains, with mean scores ranging from 2.95 to 4.02. The highest ratings were observed for hygiene knowledge (M = 4.02, SD = 1.20, 95% CI [3.88, 4.15]) and volunteer readiness (M = 3.96, SD = 1.18, 95% CI [3.83, 4.09]). In contrast, the lowest scores were reported for telemedicine (M = 2.95, SD = 1.47, 95% CI [2.78, 3.11]), simulation-based training (M = 3.32, SD = 1.36, 95% CI [3.16, 3.47]), and practical skills (M = 3.27, SD = 1.34, 95% CI [3.12, 3.43]), suggesting notable gaps in applied and experiential competencies. Given that telemedicine was the lowest-rated preparedness domain, we examined scores across educational programs. Telemedicine preparedness remained comparatively low across all programs, with no statistically significant differences observed between them [*F*(4, 291) = 2.20, *p* = 0.069, η^2^ = 0.029]. Standard deviations ranged from 1.03 to 1.47, reflecting considerable variability across responses, with narrower confidence intervals in theoretical domains and wider intervals in practice-based items.

**Figure 1.**
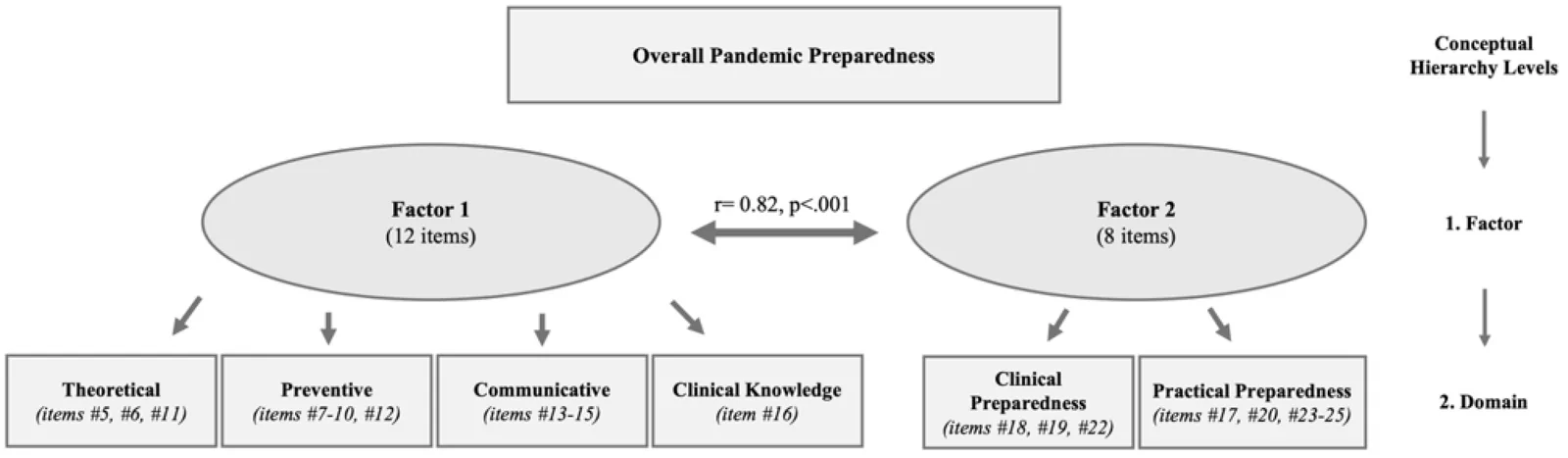
Conceptual hierarchy of pandemic preparedness factors and domains.

### 3.3. Exploratory factor analysis of preparedness dimensions

To examine the multidimensional structure of preparedness, exploratory factor analysis (EFA) was conducted on 20 Likert-scale items (Items 5–27) using principal axis factoring with direct Oblimin rotation (δ = 0). Items Q26 and Q27 were excluded because they assessed attitudes toward undergraduate education and the inclusion of pandemic-related content in curricula rather than self-perceived preparedness competencies. Sampling adequacy was excellent (Kaiser–Meyer–Olkin = 0.965), and Bartlett's test of sphericity was significant (χ^2^(190) = 5827.1, p < 0.001), confirming the suitability of the data for factor analysis. Based on the Kaiser criterion (eigenvalues > 1) and scree plot inspection, a clear two-factor solution emerged, jointly explaining 66.9% of the total variance after rotation. Factor 1 (theoretical, preventive, communicative, and clinical knowledge) comprised 12 items reflecting theoretical understanding, preventive and infection-control knowledge, public health communication and engagement, and basic clinical knowledge. Although conceptually diverse, these items clustered together in the EFA and are therefore presented as a broader knowledge-oriented dimension of preparedness. Factor 2 (clinical and practical preparedness) comprised eight items reflecting practical skills and applied response competencies, including simulation-based learning, diagnostic and emergency management, telemedicine, volunteering, and infection-control participation. Factor loadings ranged from 0.41 to 1.02, with minimal cross-loadings, and all communalities exceeded 0.30. The reported loadings represent pattern coefficients derived from an oblique (Direct Oblimin) rotation. Although one loading slightly exceeded 1.00, all communalities remained below 1.00, indicating no inadmissible solution. The two factors were strongly correlated (r = 0.82, p < 0.001, n = 296), suggesting that theoretical–public health and clinical–practical preparedness represent distinct yet related dimensions of overall readiness. Reliability analysis demonstrated excellent internal consistency for both subscales (Factor 1, α = 0.96; Factor 2, α = 0.93). Although α values exceeded 0.90, all items were retained to preserve domain coverage across preparedness dimensions (see Figure 1). Descriptive statistics based on the 1–5 Likert scale suggested moderate self-reported preparedness. Factor 1 averaged M = 3.76 (SD = 1.01, 95% CI [3.64, 3.87]), and Factor 2 averaged M = 3.42 (SD = 1.08, 95% CI [3.30, 3.54]). These results indicate that students felt more confident in their theoretical and preventive competencies than in their clinical–practical readiness. Note that the Factor 1 (M = 3.76) and Factor 2 (M = 3.42) values reported above are raw Likert-scale means (1–5). For all subsequent group comparisons and regression analyses, standardized factor scores (z-scores; M = 0, SD = 1) were used to enable comparability across subscales (see Table 1).

**Table 1.** Preparedness items by factor and domain: means (M), standard deviations (SD), and 95% confidence intervals (CI).

Factor	Domain	Item description and number	M	SD	95% CI [lower, upper]
Factor 1	Theoretical and foundational knowledge	Knowledge about pandemics (#5)	3.71	1.16	[3.58, 3.85]
		Epidemiological knowledge (#6)	3.93	1.03	[3.81, 4.04]
		Herd immunity knowledge (#11)	3.77	1.21	[3.63, 3.91]
	Preventive and infection-control knowledge	Preventive measures knowledge (#7)	3.78	1.12	[3.65, 3.91]
		Vaccination promotion knowledge (#8)	3.64	1.25	[3.50, 3.78]
		Safety measures (#9)	3.91	1.23	[3.76, 4.05]
		Hygiene measures (#10)	4.02	1.20	[3.88, 4.15]
		Infection control knowledge (#12)	3.72	1.25	[3.58, 3.86]
	Communication and public engagement	Informational and communicational measures (#13)	3.67	1.26	[3.52, 3.81]
		Raising public awareness knowledge (#14)	3.66	1.26	[3.52, 3.80]
		Increasing public engagement knowledge (#15)	3.55	1.31	[3.41, 3.70]
	Clinical knowledge	Clinical/medical knowledge (#16)	3.73	1.18	[3.60, 3.87]
Factor 2	Clinical preparedness	Diagnostic measures knowledge (#18)	3.61	1.24	[3.47, 3.75]
		Emergency medical care knowledge (#19)	3.52	1.27	[3.38, 3.67]
		Telemedicine knowledge (#22)	2.95	1.47	[2.78, 3.11]
	Practical preparedness	Simulation-based training (#17)	3.32	1.36	[3.16, 3.47]
		Volunteer readiness (#20)	3.96	1.18	[3.83, 4.09]
		Practical skill preparedness (#23)	3.27	1.34	[3.12, 3.43]
		Participation in infection-control measures (#24)	3.27	1.33	[3.12, 3.42]
		Facilitation of emergency measures (#25)	3.47	1.31	[3.33, 3.61]

### 3.4. Correlation analysis

A correlation analysis was conducted across all 20 survey items and the overall pandemic preparedness measure for the entire cohort of 296 respondents. The strongest associations were observed with practical skills (r = 0.739, p < 0.001, n = 296), participation in infection control (r = 0.734, p < 0.001, n = 296), and the ability to facilitate emergency measures (r = 0.733, p < 0.001, n = 296). Moderately strong correlations were identified for infection control knowledge (r = 0.680, p < 0.001, n = 296), informational and communicational measures (r = 0.704, p < 0.001, n = 296), diagnostic measures (r = 0.698, p < 0.001, n = 296), simulation-based learning (r = 0.685, p < 0.001, n = 296), and clinical measures (r = 0.668, p < 0.001, n = 296). Mid-range associations were found for public awareness in pandemics (r = 0.675, p < 0.001, n = 296), general knowledge of pandemics (r = 0.654, p < 0.001, n = 296), public engagement (r = 0.649, p < 0.001, n = 296), emergency medical care for patients (r = 0.644, p < 0.001, n = 296), safety measures (r = 0.634, p < 0.001, n = 296), and vaccination promotion (r = 0.630, p < 0.001, n = 296). Slightly weaker but still significant correlations were observed for preventive measures knowledge (r = 0.593, p < 0.001, n = 296), hygiene knowledge (r = 0.591, p < 0.001, n = 296), herd immunity (r = 0.590, p < 0.001, n = 296), telemedicine (r = 0.562, p < 0.001, n = 296), and readiness to volunteer in emergencies (r = 0.560, p < 0.001, n = 296), with the lowest correlation to epidemiological knowledge (r = 0.535, p < 0.001, n = 296). All correlations remained significant after Bonferroni correction (adjusted α = 0.0025; 0.05/20).

### 3.5. Group comparisons

Gender was analyzed as a three-level variable (female, male, prefer not to say) using one-way ANOVA, which showed no significant differences in preparedness across groups [F(2, 293) = 0.13, p = 0.875, η^2^ = 0.001]. Age differences were also nonsignificant [F(2, 293) = 1.01, p = 0.366, η^2^ = 0.007]. Program comparisons were performed among medicine (n = 194), dentistry (n = 86), and public health (n = 9), excluding pharmacy (n = 1) and nursing (n = 6) because of very small subgroup sizes. These comparisons revealed significant overall differences [F(2, 286) = 4.54, p = 0.011, η^2^ = 0.031]. Public health students reported the highest preparedness (M = 3.80), followed by dentistry (M = 3.44) and medicine (M = 3.36). Post hoc contrasts indicated that public health students scored higher than medicine students (mean difference = 0.44, p = 0.009, 95% CI [0.09, 0.79]), whereas the differences between public health and dentistry (mean difference = 0.36, p = 0.064), and between medicine and dentistry (mean difference = −0.08, p = 0.97) were not significant. Given the relatively small number of public health students included in the final analysis (n = 9), these findings should be interpreted cautiously. Factor 1 (theoretical, preventive, communicative, and clinical knowledge) showed no significant differences across programs [F(2, 286) = 1.99, p = 0.138, η^2^ = 0.014]. Factor 2 (clinical and practical preparedness) differed significantly across programs [F(2, 286) = 3.50, p = 0.031, η^2^ = 0.024], with public health students (M = −0.45, SD = 0.78) demonstrating higher clinical and practical preparedness compared with those in medicine (M = −0.08, SD = 1.00) and dentistry (M = 0.21, SD = 0.94). For group comparisons and regressions, factor scores were standardized (mean = 0, SD = 1) to facilitate interpretation across subscales.

### 3.6. Multiple linear regression

All models were adjusted for age, gender, and educational program as potential confounders. Sensitivity analyses excluding small subgroups (nursing, pharmacy) yielded similar results. The model predicting overall preparedness from the two factors was significant [F(2, 293) = 306.92, p < 0.001], explaining 67.7% of the variance (R^2^ = 0.677, adjusted R^2^ = 0.675, n = 296). Both Factor 1 (theoretical, preventive, communicative, and clinical knowledge) (B = 0.246, β = 0.213, 95% CI [0.101, 0.391], p < 0.001) and Factor 2 (clinical and practical preparedness) (B = 0.739, β = 0.634, 95% CI [0.593, 0.884], p < 0.001) were independent predictors of overall preparedness, with Factor 2 exerting the stronger influence on students' self-perceived readiness.

### 3.7. Logistic regression: Willingness to participate

Overall, 84.1% of students expressed willingness to participate in future pandemic responses. Among the subgroup of students who had previously participated in COVID-19 response activities (n = 79), willingness was even higher, with 97.5% indicating interest in participating in future public health emergencies. Willingness also varied slightly across educational programs. Among dentistry students (n = 86), 74.4% expressed interest in participating in future responses, and 25.6% reported no interest. Medicine students (n = 194) showed higher willingness, with 87.6% indicating interest and 12.4% reporting no interest. All respondents from public health (n = 9) and pharmacy (n = 1) expressed willingness to participate, while nursing students (n = 6) also demonstrated high willingness, with 83.3% indicating interest and 16.7% reporting no interest. The logistic regression model predicting willingness to participate from demographic and preparedness variables was significant (χ^2^(8) = 20.80, p = 0.008), explaining 11.9% of variance (Nagelkerke R^2^ = 0.119) and correctly classifying 83.7% of cases. Model fit was adequate (Hosmer–Lemeshow χ^2^(8) = 10.03, p = 0.263). Willingness was highest among public health students and lower among dentistry students (B = −0.88, p = 0.013, OR = 0.42, 95% CI [0.21, 0.83]). Age, gender, and both preparedness factors were not significant predictors.

### 3.8. Logistic regression: Receipt of extracurricular training

Receipt of extracurricular pandemic training varied by age group. Among students aged 18–22 years, 16.0% reported receiving training, compared with 25.8% of those aged 23–27 years and 46.7% of those aged 27 years or older. Overall, 20.6% of students received extracurricular training, while 79.4% did not. The logistic regression model predicting training participation from demographic and preparedness factors was significant (χ^2^(10) = 66.30, p < 0.001), explaining 30.4% of variance (Nagelkerke R^2^ = 0.304) and correctly classifying 81.1% of cases. Model fit was marginal (Hosmer–Lemeshow χ^2^(8) = 15.79, p = 0.045); patterns were robust to specification checks. Age was a significant predictor (χ^2^(2) = 7.48, p = 0.024). Students aged 23–27 years were 2.15 times more likely to have received extracurricular training (OR = 2.15, 95% CI [1.06, 4.36], p = 0.035), and those aged 27 years or older were 4.30 times more likely (OR = 4.30, 95% CI [1.16, 15.91], p = 0.029) than students aged 18–22 years. Higher scores on Factor 2 (clinical and practical preparedness) were associated with significantly increased odds of receiving extracurricular training (OR = 5.86, 95% CI [2.63, 13.05], p < 0.001). In contrast, Factor 1 (theoretical, preventive, communicative, and clinical knowledge), gender, and educational program were not significant predictors.

### 3.9. Participation in COVID-19 response

Of 296 respondents, 79 students (26.7%) reported involvement in COVID-19 management. Participation was more common among older students (χ^2^(2) = 11.67, p = 0.003, Cramer's V = 0.20), and among males (39.3%) compared with females (20.5%) and those preferring not to disclose (57.1%) (χ^2^(2) = 13.06, p = 0.001, Cramer's V = 0.21). No significant differences were observed among programs (χ^2^(4) = 3.32, p = 0.505). Among participants, involvement was highest in preventive measures (M = 3.78), followed by communication (M = 3.37) and clinical measures (M = 3.27). A Friedman test confirmed differences across domains (χ^2^(2, n = 79) = 6.44, p = 0.040). Satisfaction with contributions was moderate to high (M = 3.75), with 68.4% rating their experience positively, and 79.7% expressing willingness to share their experiences in teaching. Students who participated in the COVID-19 response demonstrated significantly higher preparedness across both domains. For Factor 1 (theoretical, preventive, communicative, and clinical knowledge), participants had higher mean scores (M = 0.27, SD = 0.99) than nonparticipants (M = −0.10, SD = 0.97) (t(294) = −2.92, p = 0.004, Cohen's d = 0.38, 95% CI [0.13, 0.64]). For Factor 2 (clinical and practical preparedness), participants likewise scored higher (M = 0.37, SD = 0.99) compared with nonparticipants (M = −0.13, SD = 0.94) (t(294) = −4.04, p < 0.001, Cohen's d = 0.53, 95% CI [0.27, 0.79]).

### 3.10. Perceptions of undergraduate education

Students endorsed the role of undergraduate education in pandemic preparedness (M = 4.04, SD = 1.09, 95% CI [3.91, 4.17], n = 296), with over 70% agreeing or strongly agreeing, and fewer than 9% disagreeing. Support for explicit inclusion of pandemic topics was stronger (M = 4.32, SD = 0.95, 95% CI [4.21, 4.44]), with 81.1% agreement. The two items were moderately correlated (r = 0.60, p < 0.001). When prioritizing reforms, students emphasized clinical and medical training (43.6%) over preventive (25.0%), theoretical (16.6%), or communication measures (14.9%) (see Figure 2).

**Figure 2.**
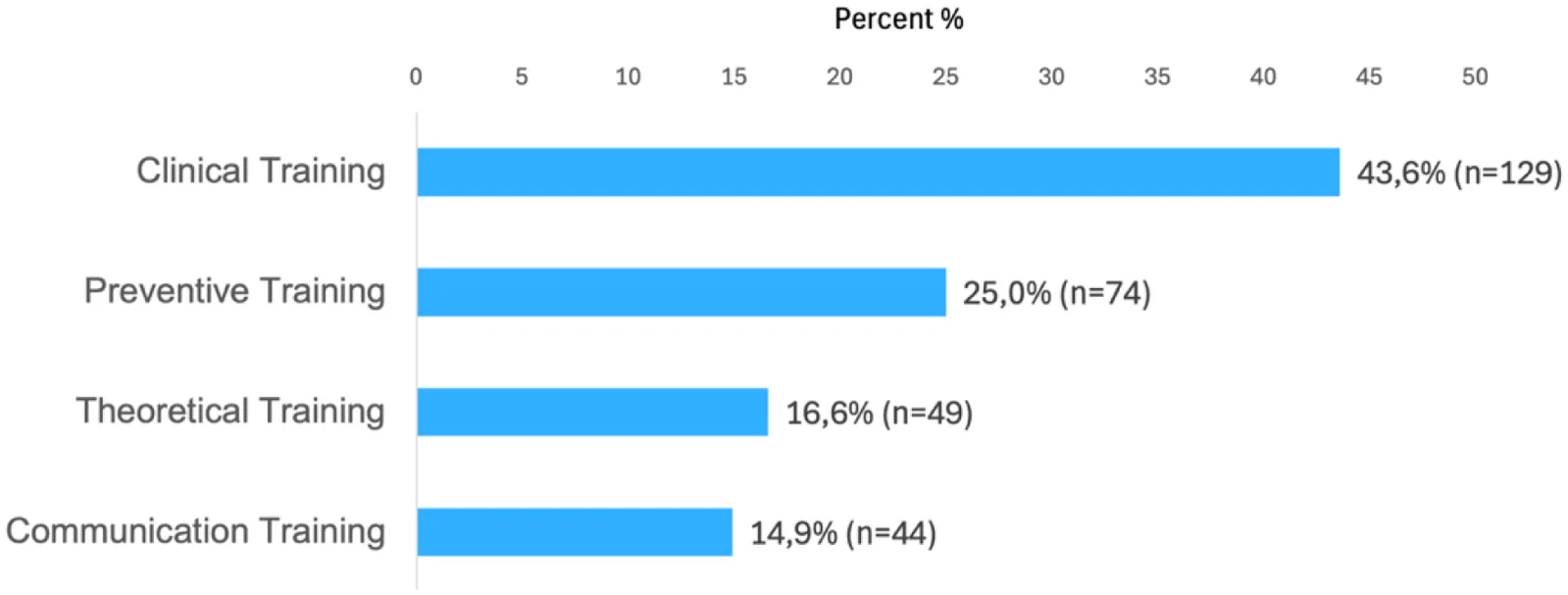
Student recommendations for curricular reform priorities (n = 296).

## 4. Discussion

### 4.1. Overview of key findings

This study identifies a gap in Georgia between students' high willingness to contribute during public health emergencies and their only moderate level of preparedness. This finding is particularly important in an upper-middle-income context characterized by workforce shortages and uneven distribution of health professionals [7]. Five years after COVID-19, pandemic preparedness remains only partially integrated into undergraduate biomedical and health sciences curricula, reinforcing earlier findings that emphasize the need for stronger practical training, simulation, and infection-control competencies [15]. Undergraduate education may play a role not only in preparing students for clinical roles but also in workforce development and emergency preparedness more broadly, functioning as a form of primary prevention by building competencies that may strengthen emergency response capacity before crises occur. In this context, the present findings provide further insight into the types of educational experiences that may contribute the most to preparedness. Practical and experiential learning components were more strongly associated with students' self-perceived preparedness than theoretical knowledge alone, highlighting the importance of moving beyond knowledge acquisition and creating opportunities for students to apply these competencies in authentic and practice-based settings.

### 4.2. Global parallels and the willingness–preparedness gap

The findings from Georgia align with international evidence describing a consistent gap between willingness and preparedness among health sciences students. Studies from South India, Indonesia, the United Arab Emirates, and China report that while a majority of students express willingness to contribute during pandemics, only a minority feel adequately prepared [16]–[19]. Systematic reviews further highlight ethical and operational concerns related to deploying students without structured training and supervision [20]. This points to a broader global challenge in aligning education with workforce needs. The high levels of willingness reported in this study may represent an opportunity to address this challenge through preparedness-focused educational initiatives. Although willingness was only partially explained by the variables included in the regression model, suggesting that additional personal, professional, and contextual factors shape students' intentions to engage, the findings indicate that preparedness is more strongly associated with practical and experiential learning than with theoretical knowledge alone. These results suggest that addressing the willingness–preparedness gap may require a shift from reactive student mobilization during crises toward the proactive integration of preparedness competencies.

### 4.3. Bridging the knowledge–practice divide

A key finding of this study is that preparedness was most strongly associated with practical and experiential domains, including practical skills, infection control participation, and emergency response engagement. In contrast, theoretical knowledge, although relatively well developed, was less predictive of overall preparedness. This pattern suggests that preparedness is shaped not only by knowledge acquisition but also by opportunities to apply knowledge in real-world contexts, which may enhance students' confidence in their ability to respond effectively during public health emergencies. These findings are consistent with evidence indicating that theoretical instruction alone is insufficient to ensure competence. Evidence from Georgia shows that students may possess general knowledge of pandemics yet lack clarity in applying this knowledge in practice [21]. Similarly, global evidence demonstrates that while online and hybrid education maintained knowledge acquisition during COVID-19, reduced clinical exposure negatively affected confidence and readiness [14]. Additional studies across different countries report similar declines in preparedness when practical training is limited [22]–[25]. Given that the outcome assessed in the present study was self-perceived preparedness, these findings may reflect students' confidence in applying knowledge during public health emergencies rather than their objectively assessed competence. As such, the lower ratings reported in practical domains should be interpreted as indicators of perceived readiness to translate knowledge into practice rather than measures of actual performance in emergency settings. From a public health perspective, this highlights the importance of structured experiential learning opportunities, such as simulation-based training, supervised clinical exposure, and participation in public health activities. Emerging evidence suggests that technology-enhanced and immersive simulation approaches may provide scalable means of strengthening applied competence and preparedness, particularly where clinical training opportunities are limited [25].

### 4.4. Student priorities and implications for curricular reform

Students in this study strongly supported the role of undergraduate education in pandemic preparedness and advocated for the inclusion of pandemic-related content in academic curricula. While they placed greater emphasis on clinical and medical training than on preventive and communication-related domains, these findings provide valuable insight into students' perceived educational needs and expectations. Nevertheless, effective pandemic preparedness requires competencies that extend beyond clinical care alone. Therefore, curricular reform should balance these preferences with the development of broader public health skills, including prevention and control measures, public health communication, and other competencies essential for a coordinated emergency response. Evidence suggests that scenario-driven education enhances applied competence and confidence [26], while interprofessional frameworks emphasize collaboration, communication, and population health approaches [27]. In resource-constrained settings, curricular reform efforts may benefit from partnerships between academic institutions, public health agencies, and healthcare organizations, allowing students to access preparedness-related expertise, training, and experiences that extend beyond the resources of a single institution.

### 4.5. Implications for health workforce development and public health systems

The findings demonstrate that participation in COVID-19 response activities and extracurricular training is associated with higher levels of preparedness. This observation is consistent with evidence from other settings, where practical experience has been associated with improved self-perceived preparedness among health sciences students [28]. Together, these findings suggest that practical and experiential learning opportunities may contribute to students' confidence in responding to future public health emergencies. However, given the cross-sectional study design, the direction of this association cannot be determined. Participation in pandemic-related activities may have enhanced preparedness, but it is also possible that students who already felt more prepared were more likely to engage in these experiences. Nevertheless, only a minority of students in this study reported receiving structured extracurricular training, indicating opportunities to expand preparedness-related learning experiences within undergraduate education. In this regard, integrating service-learning, simulation, and supervised volunteering into undergraduate education may help transform ad hoc experiences into more structured learning opportunities. Beyond their educational value, such approaches may also have broader implications for workforce development, particularly in settings facing workforce shortages and uneven distribution of health professionals. Although the present study does not directly assess workforce or health system outcomes, the findings suggest that strengthening pandemic-related competencies during undergraduate training may contribute to the development of a more prepared future health workforce.

### 4.6. From preparedness to health system resilience

The findings support a shift from reactive to proactive approaches in pandemic preparedness. Students consistently recognized the importance of education in developing readiness and supported curricular reforms that incorporate practical and applied learning. International models demonstrate that structured roles for students, participation in non-clinical public health activities, and simulation-based training can enhance preparedness and system responsiveness [29]–[32]. Adapting such approaches to the Georgian context could involve integrating interdisciplinary simulations, public health communication training, and collaboration with health authorities into existing curricula. Future efforts to expand student involvement in emergency response activities should also consider relevant ethical, legal, and supervisory frameworks, including clearly defined scopes of practice, adequate supervision, and student safety. Importantly, the findings of the study may also have relevance beyond Georgia, particularly for countries facing similar challenges related to workforce constraints and the integration of preparedness competencies into undergraduate education. While contextual differences should be considered, several of the challenges identified in this study may be shared across comparable settings. Embedding pandemic preparedness within undergraduate education may represent one critical approach to supporting preparedness among future health professionals. By aligning education with workforce needs and public health priorities, countries can better prepare future professionals to respond effectively to emerging health threats.

## 5. Conclusions

This study identifies a willingness–preparedness gap among biomedical and health sciences students in Georgia. Although most students expressed willingness to contribute during public health emergencies, preparedness remained moderate, with higher levels of self-perceived readiness associated with practical and experiential domains such as infection control, hands-on skills, and emergency response. Collectively, the findings suggest that theoretical knowledge alone may be insufficient to support students' perceived preparedness for future public health emergencies.

From a public health perspective, pandemic preparedness may benefit from being integrated more explicitly and longitudinally within undergraduate biomedical and health sciences education. In Georgia, structured experiential learning opportunities, including simulation, supervised clinical exposure, and participation in preparedness-related activities, may help strengthen students' confidence in applying preparedness-related competencies in practice. In resource-constrained settings, collaboration between institutions, public health agencies, and healthcare organizations may facilitate access to shared training resources, expertise, and preparedness activities.

The findings may also have relevance for other settings facing similar challenges in integrating preparedness into health professions education. As an upper-middle-income country where pandemic preparedness remains only partially embedded within undergraduate curricula, Georgia may serve as a useful case study for efforts to strengthen preparedness among future health professionals. Future research should extend beyond self-reported preparedness and examine how readiness develops over time. Longitudinal studies following students into the early years of professional practice may help determine whether perceived preparedness translates into competence and participation during real-world public health emergencies. Further work is also needed to identify the legal, ethical, and governance frameworks required to support student involvement in emergency response activities. Ultimately, aligning health professions education with preparedness competencies and public health priorities may help develop a workforce that is more confident in responding to future emergencies.

## 6. Limitations

Several limitations should be considered when interpreting these findings. First, the cross-sectional design precludes conclusions regarding causality or the direction of observed associations. Second, preparedness was assessed using self-reported measures rather than objective evaluations of competence. Therefore, the findings reflect students' perceptions of preparedness and may not correspond to actual performance in emergency settings. Additionally, overall preparedness was measured using a single self-assessment item, while predictor variables were derived from conceptually related self-report items. Consequently, some observed associations may have been influenced by conceptual overlap and common method variance.

Participation was voluntary, creating the possibility of self-selection bias, and social desirability bias cannot be excluded despite efforts to minimize response bias. The two-factor structure identified through exploratory factor analysis should be interpreted as exploratory and was not cross-validated in an independent sample. Furthermore, the public health, nursing, and pharmacy subgroups were small, limiting discipline-specific comparisons. Finally, due to recruitment efforts across multiple channels, an overall response rate could not be calculated. Despite these limitations, the study provides important insights into pandemic preparedness and willingness to participate in future public health emergencies among biomedical and health sciences students in Georgia.

## Use of AI tools declaration

The authors declare that generative artificial intelligence (AI) tools (ChatGPT) were used solely for grammatical and stylistic refinement of the manuscript. All study design, data collection, statistical analysis, interpretation of results, and scientific conclusions were conducted entirely by the authors. The authors take full responsibility for the accuracy, integrity, and originality of the content.


