# Supplementary material for: Health workforce readiness for future pandemics: Evidence from undergraduate health sciences students in Georgia
Source: AIMS Public Health. 2026 Aug 6;13(3):831–48. doi: 10.3934/publichealth.2026044 (PMC13684079; doi:10.3934/publichealth.2026044)
Supplement: Supplementary file 1 [file publichealth-13-03-044-s001.pdf]

Public Health

AIMS Public Health, 13(3): 831–848.
DOI: 10.3934/publichealth.2026044
Received: 23 March 2026
Revised: 21 June 2026
Accepted: 06 July 2026
Published: 06 August 2026

http://www.aimspress.com/journal/aimsph

Research article

Health workforce readiness for future pandemics: Evidence from undergraduate health sciences students in Georgia

Dea Goderdzishvili* and Elza Nikoleishvili

Public Health and Healthcare Management Department, School of Health Sciences, The University of Georgia, Tbilisi, Georgia

* **Correspondence:** Email: dea.goderdzishvili@ug.edu.ge; deagoder@gmail.com; Tel: +995595 975010.

Supplementary Table 1. Pandemic preparedness and readiness questionnaire.

Item No.	Question or item	Responses
Section 1: Demographic data		
1	Gender	Female
		Male
		Prefer not to say
2	Age	18–22 years
		23–27 years
		27+ years
3	Your university (write the name of your university)	
4	Educational program	Medicine
		Dentistry
		Pharmacy
		Public health
		Nursing
Section 2: Curricular pandemic preparedness		
5	To what extent do you agree with the following statement:	Strongly disagree
	"My university's curriculum provides the acquisition of knowledge about the course, development and the expected outcomes and consequences of pandemics"	Somewhat disagree
		Neutral / Neither agree nor disagree
		Somewhat agree
		Strongly agree

Continued on next page

Item No.	Question or item	Responses
6	To what extent do you agree with the following statement:<br>"I have basic knowledge of epidemiological concepts related to pandemics"	Strongly disagree<br>Somewhat disagree<br>Neutral / Neither agree nor disagree<br>Somewhat agree<br>Strongly agree
7	To what extent do you agree with the following statement:<br>"Through the curriculum of my educational program, I acquired the appropriate theoretical knowledge to participate in various preventive measures during pandemics"	Strongly disagree<br>Somewhat disagree<br>Neutral / Neither agree nor disagree<br>Somewhat agree<br>Strongly agree
8	To what extent do you agree with the following statement:<br>"Through the curriculum of my educational program, I acquired the appropriate knowledge to implement vaccination promotion measures during pandemics"	Strongly disagree<br>Somewhat disagree<br>Neutral / Neither agree nor disagree<br>Somewhat agree<br>Strongly agree
9	To what extent do you agree with the following statement:<br>"Through the curriculum of my educational program, I acquired the appropriate knowledge to implement safety measures during pandemics"	Strongly disagree<br>Somewhat disagree<br>Neutral / Neither agree nor disagree<br>Somewhat agree<br>Strongly agree

Continued on next page

Item No.	Question or item	Responses
10	To what extent do you agree with the following statement:	Strongly disagree
	"Through the curriculum of my educational program, I acquired the appropriate knowledge to implement hygiene measures during pandemics"	Somewhat disagree
		Neutral / Neither agree nor disagree
		Somewhat agree
		Strongly agree
11	To what extent do you agree with the following statement:	Strongly disagree
	"Through the curriculum of my educational program, I acquired the appropriate knowledge regarding the aspects of the population (so-called "herd") immunity during pandemics"	Somewhat disagree
		Neutral / Neither agree nor disagree
		Somewhat agree
		Strongly agree
12	To what extent do you agree with the following statement:	Strongly disagree
	"Through the curriculum of my educational program, I acquired the appropriate knowledge to implement infection controls measures during pandemics"	Somewhat disagree
		Neutral / Neither agree nor disagree
		Somewhat agree
		Strongly agree
13	To what extent do you agree with the following statement:	Strongly disagree
	"Through the curriculum of my educational program, I acquired appropriate knowledge to implement informational and communicational measures during pandemics"	Somewhat disagree
		Neutral / Neither agree nor disagree
		Somewhat agree
		Strongly agree

Continued on next page

Item No.	Question or item	Responses
14	To what extent do you agree with the following statement:<br>"Through the curriculum of my educational program, I acquired the appropriate knowledge to implement proper measures to raise public awareness during pandemics"	Strongly disagree<br>Somewhat disagree<br>Neutral / Neither agree nor disagree<br>Somewhat agree<br>Strongly agree
15	To what extent do you agree with the following statement:<br>"Through the curriculum of my educational program, I acquired the appropriate knowledge to increase public engagement during pandemics"	Strongly disagree<br>Somewhat disagree<br>Neutral / Neither agree nor disagree<br>Somewhat agree<br>Strongly agree
16	To what extent do you agree with the following statement:<br>"Through the curriculum of my educational program, I acquired the appropriate knowledge to carry out clinical/medical measures during pandemics"	Strongly disagree<br>Somewhat disagree<br>Neutral / Neither agree nor disagree<br>Somewhat agree<br>Strongly agree
17	To what extent do you agree with the following statement:<br>"The simulation training (emergency aid, emergency situation training, etc.) provided by the curriculum of<br>my educational program, helped me to actively participate in preventive-treatment measures during the COVID-19 pandemic"	Strongly disagree<br>Somewhat disagree<br>Neutral / Neither agree nor disagree<br>Somewhat agree<br>Strongly agree

Continued on next page

Item No.	Question or item	Responses
18	To what extent do you agree with the following statement:<br>"Through the curriculum of my educational program, I acquired the appropriate knowledge to carry out diagnostic measures during pandemics"	Strongly disagree<br>Somewhat disagree<br>Neutral / Neither agree nor disagree<br>Somewhat agree<br>Strongly agree
19	To what extent do you agree with the following statement:<br>"Through the curriculum of my educational program, I acquired the appropriate knowledge to provide emergency medical care to patients during pandemics"	Strongly disagree<br>Somewhat disagree<br>Neutral / Neither agree nor disagree<br>Somewhat agree<br>Strongly agree
20	To what extent do you agree with the following statement:<br>"I am ready, as a volunteer, to actively participate in the management and monitoring of emergency<br>situations in the country"	Strongly disagree<br>Somewhat disagree<br>Neutral / Neither agree nor disagree<br>Somewhat agree<br>Strongly agree

Continued on next page

Item No.	Question or item	Responses
21	Please indicate in which subject you gained knowledge and information about pandemics (you<br>can choose more than 1 subject)	Public health<br>Epidemiology<br>Public health management<br>Immunology<br>Infectious diseases<br>Emergency medicine<br>Internal medicine<br>Preventive medicine<br>Control of infectious diseases<br>Hygiene control<br>Emergency situations<br>First aid<br>None<br>Other
22	To what extent do you agree with the following statement:<br>"Through the curriculum of my educational program, I acquired the appropriate knowledge about the<br>importance of medical technology, such as Telemedicine."	Strongly disagree<br>Somewhat disagree<br>Neutral / Neither agree nor disagree<br>Somewhat agree<br>Strongly agree

Continued on next page

Item No.	Question or item	Responses
23	To what extent do you agree with the following statement:<br>“The curriculum of my educational program adequately prepared me in terms of practical skills needed to respond to pandemics”	Strongly disagree<br>Somewhat disagree<br>Neutral / Neither agree nor disagree<br>Somewhat agree<br>Strongly agree
24	To what extent do you agree with the following statement:<br>“The curriculum of my educational program adequately prepared me to adequately participate in infection<br>control measures during pandemics”	Strongly disagree<br>Somewhat disagree<br>Neutral / Neither agree nor disagree<br>Somewhat agree<br>Strongly agree
25	To what extent do you agree with the following statement:<br>“The curriculum of my educational program adequately prepared me in terms of facilitating the effective implementation of emergency measures during pandemics (e.g. quarantine, ‘lock-down’)”	Strongly disagree<br>Somewhat disagree<br>Neutral / Neither agree nor disagree<br>Somewhat agree<br>Strongly agree
26	To what extent do you agree with the following statement:<br>“Undergraduate education plays an important role in the responding to pandemic preparedness and<br>response management issues”	Strongly disagree<br>Somewhat disagree<br>Neutral / Neither agree nor disagree<br>Somewhat agree<br>Strongly agree

Continued on next page

Item No.	Question or item	Responses
27	To what extent do you agree with the following statement:<br>“Undergraduate education curriculum should include topics related to pandemics, such as prevention, informational-communicational and effective management of clinical measures”	Strongly disagree<br>Somewhat disagree<br>Neutral / Neither agree nor disagree<br>Somewhat agree<br>Strongly agree
28	Based on the knowledge gained from your university curriculum how would you rate your preparedness to respond to pandemics?	Fully unprepared<br>Somewhat unprepared<br>Neutral<br>Somewhat prepared<br>Fully prepared
29	Have you received extracurricular preparatory training in the management measures of the COVID-19 pandemic?	Yes<br>No
30	Have you participated in the management measures of the COVID-19 pandemic?	Yes<br>No
Section 3: COVID-19 response participation experience		
31	How involved were you in the preventive measures during the COVID-19 pandemic? (e.g. participation in the vaccination program, promotion of compliance with quarantine measures)	Not involved at all<br>Slightly involved<br>Moderately involved<br>Very involved<br>Extremely involved

Continued on next page

Item No.	Question or item	Responses
32	How involved were you in the informational-communicational measures during the COVID-19 pandemic? (e.g. working in a call center, spreading information about vaccinations or social distancing measures)	Not involved at all<br>Slightly involved<br>Moderately involved<br>Very involved<br>Extremely involved
33	How involved were you in the clinical/medical measures during the COVID-19 pandemic? (e.g. working as a doctor's or nurse's assistant, collecting laboratory swabs and samples)	Not involved at all<br>Slightly involved<br>Moderately involved<br>Very involved<br>Extremely involved
34	How satisfied are you with the actions you have taken in response to the COVID-19 pandemic?	Very dissatisfied<br>Dissatisfied<br>Neutral<br>Satisfied<br>Very satisfied
35	Are you interested in sharing the experience gained in the management of the COVID-19 pandemic for the purposes of teaching/training undergraduate students?	Yes<br>No
Section 4: Future Pandemic Preparedness and Readiness		
36	Are you interested in participating in future pandemic preparedness and response activities?	Yes<br>No

Continued on next page

Item No.	Question or item	Responses
37	In your opinion, what educational and practical measures can your university implement to promote possible future pandemic preparedness and readiness?	Strengthening theoretical teachings about pandemics in the educational curriculum
		Conducting additional educational trainings for implementation of preventive measures
		Conducting additional educational trainings for implementation of informational-communicational measures
		Conducting additional educational trainings for implementation of clinical/medical measures
		Other

AIMS Press

© 2026 the Author(s), licensee AIMS Press. This is an open access article distributed under the terms of the Creative Commons Attribution License (http://creativecommons.org/licenses/by/4.0)
